# Tool use ability depends on understanding of functional dynamics and not specific joint contribution profiles

**DOI:** 10.3389/fpsyg.2014.00306

**Published:** 2014-04-23

**Authors:** Ross Parry, Gilles Dietrich, Blandine Bril

**Affiliations:** ^1^Sorbonne Universités, UPMC Univ Paris 06, UMR_S 975, CRICMParis, France; ^2^Inserm, U 1127, CRICMParis, France; ^3^UFR STAPS, Université Paris DescartesParis, France; ^4^Groupe de Recherche Apprentissage et Contexte, Ecole des Hautes Etudes en Sciences SocialesParis, France

**Keywords:** tool use, motor learning, motor equivalence, synergy, expertise, mechanical constraints, stone knapping, mechanical reasoning

## Abstract

Researchers in cognitive neuroscience have become increasingly interested in how different aspects of tool use are integrated and represented by the brain. Comparatively less attention has been directed toward tool use actions themselves and how effective tool use behaviors are coordinated. In response, we take this opportunity to consider the mechanical principles of tool use actions and their relationship to motor learning. Using kinematic analysis, we examine both functional dynamics and joint contribution profiles of subjects with different levels of experience in a primordial percussive task. Our results show that the ability to successfully produce stone flakes using the Oldowan method did not correspond with any particular joint contribution profile. Rather, expertise in this tool use action was principally associated with the subject's ability to regulate the functional parameters that define the task itself.

## Introduction

The study of human tool use necessitates the observation of interactions with the surrounding environment. Indeed, the very notion of tool use itself implies the appropriation of an object (external to the organism) from the environment. More importantly though, the purpose of tool use generally is to extend one's ability to effect change upon the environment (Leroi-Gourhan, 1964; Baber, 2006). Any instance of tool use behavior should therefore be regarded primarily as a goal directed action and thus can only be effectively evaluated in relation to the demands of the situation or task at hand (Bril et al., 2010; Nonaka et al., 2010).

**Figure 1:**
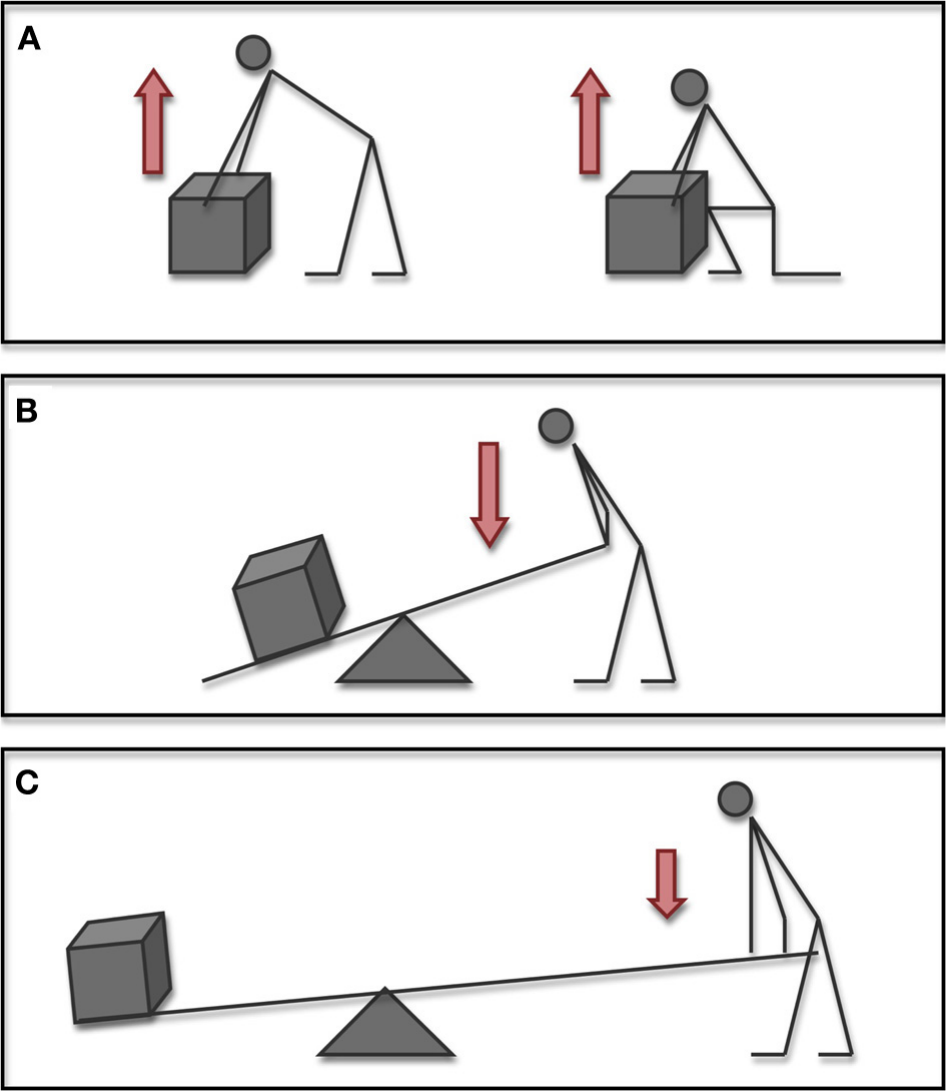
**In order to successfully learn goal directed actions, the actor must learn the functional dynamics of the task**. Whilst different movements or tools may be used, each person is obliged to learn how body-object-environment relationships combine to satisfy task constraints. In these examples, the weight of the box (mass × gravity), indicated by the white arrow remains constant. The force applied by the actor (indicated here by the red arrow) may however vary as conditions change. **(A)** In the example of lifting a box, an individual may use any number of postural combinations. Successfully lifting the box requires only that the person generates enough force to overcome the mass of the box with the combined effect of gravity. **(B)** Upon introduction of a simple tool such as a lever, the dynamics of the task change considerably. **(C)** Adjusting relationships between tool-person-environment configurations may significantly change functional dynamics of the situation. Here this may be due to adjusting the location of the load and pivot, or alternatively by increasing the size of the lever.

Determining the efficacy of a tool use action however, is not as simple as it may appear. Like most other motor tasks, an effective tool use action may be generated using a multitude of different postural combinations (refer to Figure 1). The question of how the brain goes about choosing one particular movement is a central theme in the study of motor control and is commonly referred to as the motor equivalence problem (Bernstein, 1967). In effect, successful achievement of the desired outcome in any motor problem requires only that the actor satisfy the constraints of the task at hand. It is thus the mechanics of the task that impose the characteristics of the action (Bril et al., 2009, 2010). Adaptive behavior then emerges as the nervous system learns to exploit the mechanical properties that exist in the different body-environment configurations (Bernstein, 1967; Chiel and Beer, 1997).

When compared with other motor tasks though, the distinguishing feature of tool use behavior is the incorporation (by the actor) of an external device in order to mediate the physical interaction constitutive of the goal (Preston, 1998; Bongers et al., 2004; Baber, 2006). It is a permutation that effectively entails several consequences. On one hand, the introduction of the tool adds greater complexity to the existing body-environment system. And beyond the evident addition of the physical characteristics of the tool itself, each and any variation of the tool's relationship to the body and to the environment may have significant repercussions upon task performance (van Leeuwen et al., 1994; Bongers et al., 2004). In purely mechanical terms, introducing a greater number of degrees of freedom to the system necessitates more sophisticated methods of control. On the other hand, modifying the dynamics of the system can afford potential benefits, not the least of which may be greater precision or mechanical advantage. In this respect, tool use may be considered a game of functional dynamics, of learning and mastering a complex system of mechanical conditions in a body-task-environment interaction to the desired effect (Roux et al., 1995; Smitsman, 1997).

The ability to perceive and manage varying complexity of physical interactions is thus fundamental. It defines adaptive tool use behavior. Remarkably though, very few studies of tool use place emphasis upon these phenomena as part of their experimental design, focusing rather on action plans and neural representations. Frequently, studies on tool use behavior have either sought to eliminate the need for subjects to negotiate the physical interactions for which that tool is conceived, (e.g., pantomime, naming, or recognition of tools, observing tool action, imagining tool use) or otherwise significantly reduced the degrees of freedom existing between actor and tool (see among others Choi et al., 2001; Kellenbach et al., 2003; Johnson-Frey et al., 2005; Lewis, 2006; Goldenberg et al., 2007; Stout et al., 2008; Peeters et al., 2009, 2013; Ramayya et al., 2010; Massen and Sattler, 2012).

Importantly, several recent papers have confronted the cognitive processes involved in understanding the physical interactions involved in tool use. In examples of clinical studies, Goldenberg and Hagmann (1998) and Hodges et al. (1999) proposed that a form of “mechanical reasoning” may support functional tool use, enabling an individual to determine appropriate actions by means of comparison between structural properties of the objects (both tool and target material) with respect to task demands. More recently, Osiurak et al. (2009) expanded upon this work using the notion of “technical reasoning”—a capacity that presents as being distinct from those involved in object representation. In all cases though, the basis of these types of mechanical reasoning processes have been examined in the context of tool selection or by classification of the action demonstrated (e.g., correct/incorrect; object error/action error). As yet these studies have not yet been extended to include the use of quantitative evaluation (by means of kinetic analysis, for example) of subject ability to control the physical interactions critical to the task.

Undoubtedly, these technical and mechanical reasoning frameworks above have proven themselves to be rather informative, most notably in the study of apraxia. Still, the interest of these models has been found primarily in their utility for determining the roles played by the various cognitive processes involved in tool use (Goldenberg and Spatt, 2009; Osiurak et al., 2009). Indeed for the most part in tool use research, the human ability to engage in complex tool use has been perceived predominantly as a function of cognitive capacity. The overwhelming prevalence of research methods focused primarily upon cognitive and cerebral activity does seem to be somewhat at odds with the problematic itself. After all, unlike certain other skills that frequently occur as exclusively internal cognitive processes (e.g., planning, recall, or arithmetic), tool use does not “happen in the brain.” The tool use action itself may be seen to embody the actor's capacity to perceive relevant stimulus and coordinate an efficient response with respect to the situation at hand (Preston, 1998; Baber, 2006; Bril et al., 2009). As such, functional approaches to the analysis of tool use behavior may provide particularly rich information regarding the cognitive abilities of actor (Bril et al., 2009).

Accordingly, it is imperative to recognize that that locus of control does not rest exclusively in the brain. Effective tool use necessitates organization across an exceptionally intricate system spanning both the central and peripheral nervous systems. More than just a question of internal representation, adaptive tool use is equally a question of dexterity (Bernstein, 1996). To focus exclusively upon mechanisms for transforming sensory representations of the body and environment into motor programs is thus insufficient for explaining the complexity of tool use behavior. Moreover, the division made between cognitive and motor aspects of performance implied by such methods appears to be more an academic convenience than a physiological reality (Newell, 1991; Summers and Anson, 2009). Indeed, the very notion of the motor program, though an ever-present paradigm in both research and clinical perspectives, is obscure at best and no real consensus exists on whether it should be regarded as a literal or metaphorical concept (Newell, 1991; Morris et al., 1994; Ostry and Feldman, 2003; Latash, 2008a; Summers and Anson, 2009).

The study of motor control thus provides a rather privileged manner for evaluating cognitive and neural bases of tool use. Through the study of motor control, one may effectively see what is controlled in terms of mechanical principles (e.g., velocity, force, energy). Further to this, motor control allows the observer to see how the action is controlled, most commonly in the form of kinematic organization. Looking at a series of tool use actions in this manner provides valuable insight into how the nervous system as a whole prioritizes or controls different aspects of the action. This is, in essence, the same logic used by Bernstein in some of the earliest studies of motor control in tool use (reviewed by Latash, 2000; Biryukova and Bril, 2002). Conducted during the 1920s and at the height of Taylorism, Bernstein's studies had been organized under the direction of the Soviet Ministry for Scientific Labor Organization. Their purpose had been to facilitate the standardization of labor techniques and thereby increase worker efficiency. When analyzing the hammering techniques of expert blacksmith's however, Bernstein made a rather remarkable observation. Although it had been expected that variability in joint contributions would be indicative of poor hammer control, this was not the case. Rather, despite considerable variability of joint angle contributions through the striking arm, expert blacksmiths exhibited minimal variability of the hammer's working point trajectory.

The results of these experiments highlight in a simple yet elegant manner some interesting points regarding expert movement and neural organization. Evidently, in the case of these expert subjects, the nervous system did not seek to exploit any unique movement pattern, a variety of functionally equivalent movements were used to comparable effect. For Bernstein, it seemed unlikely that the brain would specifically prescribe different kinematic and kinetic profiles upon each trial, with individually programmed joint trajectory and muscle activation patterns. He concluded that during these expert movements, the ensemble of joints comprising the multi-segmental effector system was compensating for variability arising from each individual articulation.

Today the terms “synergy” and “coordinative structure” are commonly used to describe this functionally specific organization of neural, muscular, and skeletal elements evoked in Bernstein's observations (Latash, 2008b; Kelso, 2009). It is maintained that the arrangement of motor apparatus in such a way permits the highly flexible and responsive movement characteristic of dexterous tool use. Assembled across the nervous system as the situation or context evolves, the synergy facilitates sensory and mechanical feedback—effectively modulating network activity so that the task specific objectives may be stabilized. This theory that motor control is organized by these coordinative structures is also consistent with physiological literature. For example, it has been demonstrated that descending tract activity is in fact unable to directly prescribe muscle activity in terms of torque or trajectory. The central commands instead appear to regulate postural and movement responses by changing threshold values of muscle length (Matthews, 1959; Ostry and Feldman, 2003; Houk and Rymer, 2011). It has been argued that the existence of synergies is evidenced by the exceptionally rapid adaptation of movement in response to perturbation during goal directed activity (Kelso, 2009). The Uncontrolled Manifold Hypothesis (UCM; Scholz and Schöner, 1999) provides a method of measuring the coordinative structure by separating the movement variability that does not affect the performance outcome (compensated variability) from the movement variability that does compromise one's ability to satisfy the task requirements (non-compensated variability).

Whilst the blacksmiths of Bernstein's early work on percussive tool use indicated that experts tend to exploit the abundant degrees of freedom at their disposal in actual tool use activity, the relationship between functional dynamics and movement variability is less clear during other phases of motor learning. In a recent theoretical article, Latash (2010) described a novel view on stages of motor learning using this principle of synergies. It was proposed that initial stages of learning, obliged the actor to explore functional dynamics of the task at hand to allow for the discovery of effective movement parameters. With increased experience, task performance would then become stabilized as synergies became more robust—thus allowing greater flexibility of movement as the neuromotor system became more adept at regulating the mechanical conditions necessary for successful task performance. Finally, once movement synergies reached a stage where uncompensated variability could not be further reduced, their composition may be altered in order to optimize other relevant factors secondary to task performance, favoring for example, energy conservation or the aesthetic features of movement.

In this paper we use an experimental protocol to explore how movement synergies develop in tool use. This will be done through the observation of coordinative structure at different levels of expertise. We propose here that tool use capacity is first and foremost a learned ability to manipulate the functional dynamics of the task at hand. Given this, our analysis will focus upon subject ability to satisfy task constraints and its relationship to kinematic movement patterns.

The data presented here adds to the existing body of work based around the technique of stone knapping. It is a technique that involves the removal of stone flakes from a flint core and was widely employed by prehistoric man in the production of edged cutting tools. Certain knapping techniques continue to be used today, in the production of architectural flint and artisanal crafts for example. Given that stone knapping provides the earliest known evidence of human tool use and tool production, it is often considered to reflect both cognitive and manual skills that distinguish human tool use abilities from those of other species. Previous studies have linked stone knapping to the evolution of anatomical and biomechanical properties of the upper limb (Marzke and Marzke, 2000; Rolian et al., 2011; Williams et al., 2012); the expansion of cortico-cerebellar circuitry supporting motor control (Bril et al., 2012) and the acquisition of the cognitive capacities supporting language and communication (Toth et al., 1993; Stout et al., 2008; Stout and Chaminade, 2012; Uomini and Meyer, 2013).

**Figure 2:**
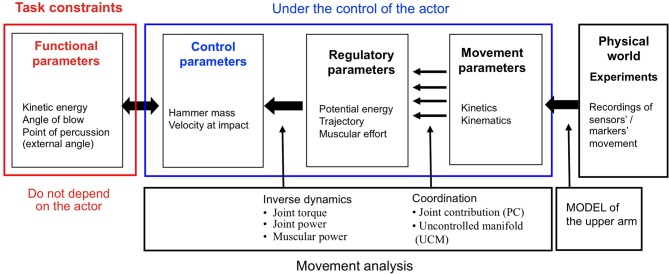
**Framework for the study of percussive tool use actions**. Successful task performance is dependent upon satisfying task constraints. The experiment presented here used a series of sensors to record upper limb movement. Reconstruction of this data using a biomechanical modeling process then enabled the study of how subjects controlled the various parameters involved in the percussive task (adapted from Bril et al., 2010, 2012).

In certain respects, the removal of a stone flake is similar to other percussive tasks such as driving a nail into wood, hitting a golf ball, or breaking the hard shell of a nut. All require the use of forceful, striking movements to achieve the goal at hand. What distinguishes one of these tasks from any other though are the objectives of the activity, the materials involved and the functional dynamics of each situation. Here we use the framework developed by Bril and colleagues (Bril et al., 2009, 2010, 2012; Nonaka et al., 2010; Rein et al., 2013) in their previous studies on the mastery of percussive techniques as a practical framework for defining the characteristics of the task and the performance of the actor (see Figure 2).

In this model, the task constraints are the conditions necessary to effectuate the desired goal. To satisfy task constraints, the actor must generate specific values of functional parameters (kinetic energy, angle of blow, and point of percussion). The actor may do this by using any one of a variety of mutually dependent combinations of control parameters (hammer mass, velocity at impact). In turn, a multitude of potentially valid strategies (potential energy, trajectory, muscular effort) are at the disposal of the actor as he attempts to regulate the relationship between these combinations of control parameters. Finally, given that the number of degrees of freedom defining the task constraints is fewer than the number found in the multi-segmental effector system, there exist an infinite number of combinations of movement parameters (kinetics, kinematics, muscle control) that could serve as valid motor solutions. This framework facilitates the study of percussive tasks by highlighting interplay between the complexity of a percussive task, the strategy chosen by the actor, and the coordination of the movement during the performance of the action.

**Figure 3:**
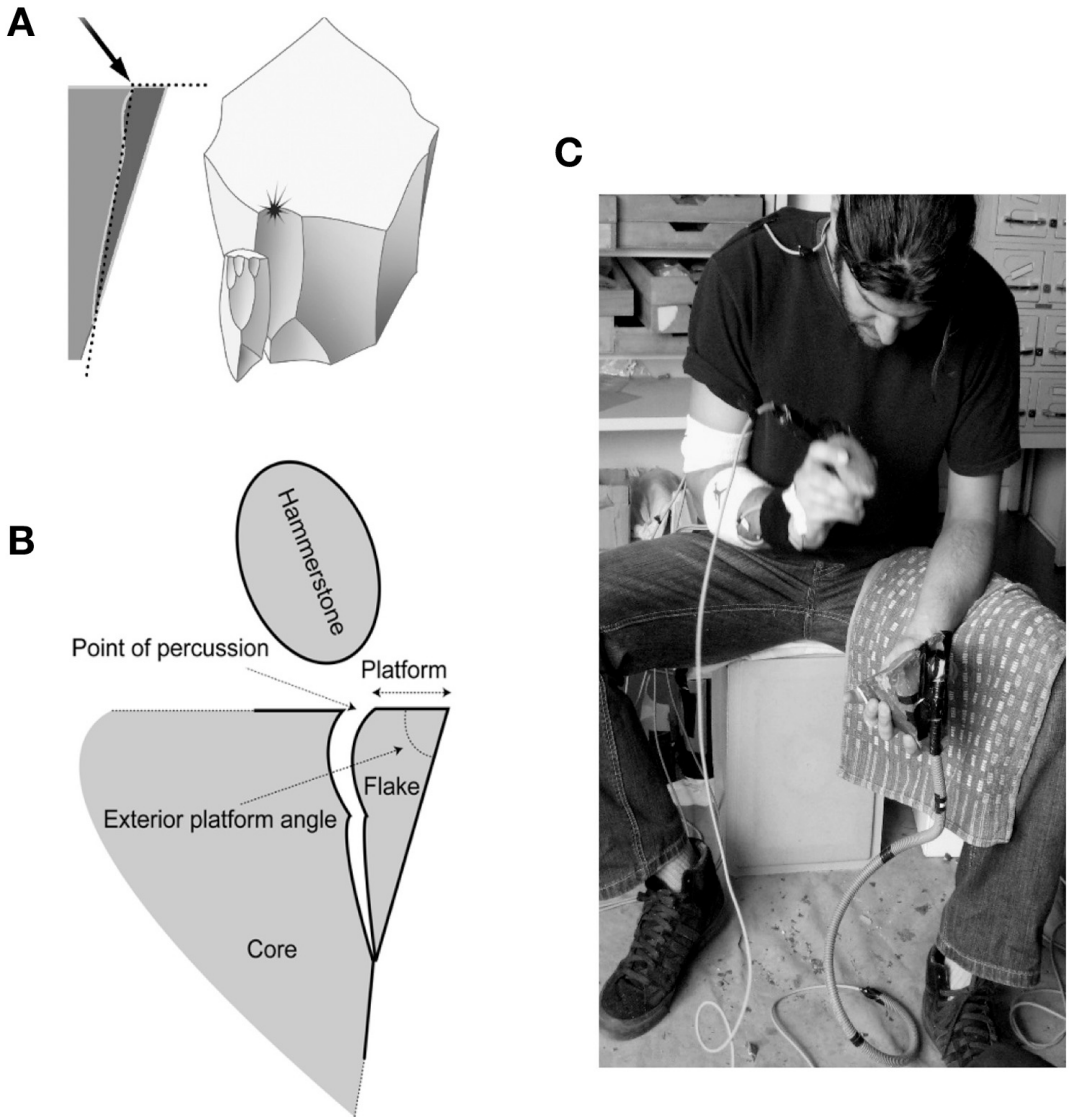
**The stone flaking process. (A)** Conchoidal fracture results from an angle of percussion of approximately 40–50° and an exterior angle of approximately 70–80° (adapted from Pelegrin, 2005, with permission); **(B)** Terminology used to describe the mechanics of stone flaking. (adapted from Bril et al., 2012); **(C)** An expert subject in the process of removing a stone flake under experimental conditions. The core is positioned using the left hand while the strike is delivered using the hammerstone in the subjects right hand. Movement sensors can be seen on the subject's arm as well as the working materials. This experiment analyzed movements from the highest position of the striking hand through to the completion of the strike.

As with other fine grain materials such as glass, cornelian and quartz, the intentional shaping, or reduction or a flint core is made possible through conchoidal fracture (Roux et al., 1995). Successfully removing a stone flake by this action is dependent upon relationships between several variables; the external platform angle, the point of percussion, the angle of the blow and the kinetic energy delivered to the point of impact (see Figure 3). The conchoidal fracture is contrasted with “split breaking,” which can occur independently of these other variables upon the application of a sufficiently large force. The removal of flakes by split breaking offers very limited control over the form and number of flakes produced (see Pelegrin, 2005; Bril et al., 2012 for further discussion).

In practice, producing a stone flake of a pre-determined form is by no means a trivial accomplishment; it requires highly attuned perceptual-motor capacities. For example, in the research presented by Nonaka et al. (2010), participants of varying levels of experience in Oldowan stone knapping techniques were required to trace an outline indicating the dimensions of the flake they intended to produce prior to each attempt. Only those subjects having extensive knapping experience demonstrated the capacity to reliably predict and control the flake removal process, effectively revealing their expert appreciation for the higher order relationships existing between the multiple functional parameters at play.

As part of the experiments presented by Bril et al. (2010), participants in a series of stone knapping activities were required to use hammerstones with varying mass. Several particularly interesting observations were made through the course of the analysis. Firstly, only expert knappers were able to adapt their movements in such a way that left the resulting kinetic energy unaltered between hammer conditions, proving their remarkable sensitivity to this key functional parameter. Secondly, whereas novice and intermediate subjects tended to compensate for lower hammer masses by increasing the muscular force they applied, expert subjects maintained resultant kinetic energy by increasing trajectory length and thereby the potential energy upon initiation of the movement. In other words, when adapting to different control parameters, experts sought motor solutions that harnessed external forces, namely that of gravity.

The movement parameters involved in stone knapping tasks have also drawn the attention of several recent studies. In a paper by Williams et al. (2010), kinematic analysis revealed a proportionately high level of movement at the wrist in four beginner/novice stone knappers. Together with a proximal-distal sequencing pattern, the high level of wrist activity was judged to be advantageous in developing greater accuracy and velocity. Conversely, in a study with four experts and eight novices, Rein et al. (2013) observed that the elbow joint provided a greater contribution to the knapping task than both the shoulder and wrist joints. Reflecting the findings of Bril et al. (2010), expert subjects in this experiment were also observed to exhibit smaller hammer velocities than subjects of the novice group.

As part of the data analysis process, Rein et al. (2013) also attempted to characterize movement variability using the UCM and determined that stone knappers coordinate their movement to minimize the variability of the hammerstone's working point trajectory (one should be mindful however, that the UCM analysis of this study was limited to movement characteristics pertaining to the striking arm and did not test hypotheses regarding control as it related to the functional parameters themselves). And while novice subjects did exhibit greater variability of working point trajectory than experts, this fact may be more a symptom of their poor understanding of functional parameters than an inability to coordinate the movement itself. Overestimating the importance of velocity at the time of impact will inevitably have negative consequences upon precision (Fitts, 1954; see also Domkin et al., 2002, for another example of this effect in a UCM analysis).

In addition to studies on Oldowan stone flaking, stone knapping by counterblow, a technique used by artisanal craftsmen in India, has also been the subject of several studies on complex tool use behavior. In relation to the movement capacities of these artisans, Biryukova and Bril (2008) found the kinematic patterns of a group of expert subjects to be strikingly individual. More intriguing still was the fact that the most expert and versatile subject amongst the craftsmen demonstrated far greater joint angle contribution variability than other subjects. They concluded that the number of joints involved and similarly the potential number of effective joint angle contributions available to a subject increased as a function of skill.

In contrast to the prior studies, the following experiment incorporates actors of varying levels of expertise. Included are subjects having no prior experience at all on the set task, nor an academic appreciation of stone tool techniques (referred to hereon as uninitiated subjects). Other subjects of this study present with a varying range of skill and experience in stone knapping. This design hence permits study of the stone flaking action at novice, intermediate, and expert levels. Instead of exploring movement performance through tools for measuring central tendency, this study will place the emphasis on the features of a series of individual movements. In doing so, we intend to observe if certain movement patterns or strategies are typical at a given stage of expertise in tool use.

Here, tool use is considered primarily as a goal directed activity, fundamentally defined by task specific mechanical principles. We suggest that rather than seeking to learn a specific movement, the nervous system seeks to learn the action (Bernstein, 1996; Reed and Bril, 1996) through exploration of the functional dynamics of the body-tool-environment system. As such, we hypothesized that numerous kinematic patterns would prove effective in the stone flaking task. In other words, we anticipated that successful tool use actions would not be characterized by any particular kinematic profile.

Our second hypothesis was that kinematic movement variability would fluctuate according to a subject's sensitivity to functional dynamics. It was expected that subjects having no prior experience on this novel task (the uninitiated group) would employ highly variable movement patterns as they would be obliged to explore the dynamics of this complex tool use activity. Having discovered a limited set of body-tool-environment configurations in satisfying task constraints, novice, and intermediate subjects were expected to have more regular kinematic movement profiles. Lastly, expert tool use performance was expected to demonstrate a high level of sensitivity to the functional parameters during the stone flaking task (Bril et al., 2010; Nonaka et al., 2010). We anticipated that these subjects would have more variable kinematic movement profiles as robust synergies ensured the stability of functional parameters through the flexible covariation of upper limb segments.

## Materials and methods

### Participants

A total of 19 human subjects (8 males, 11 females) participated in this study. The mean age of the sample was 35.3 years (median 28 years; standard deviation 14.5 years; range 23–71 years). The absence of pathology impacting upon upper limb function was a condition for participation in this experiment. Only one subject, a flint worker by profession, was remunerated for his participation. All other subjects were unpaid volunteers. The majority of the subjects having a background understanding of lithic tool production were recruited through academic institutions in the Paris region. The flint-working professional (P18-JL) and one experienced hobbyist (P19-BM) were recruited separately through existing professional relationships. Subjects having no knowledge or experience in stone knapping techniques (the uninitiated group) were sourced from visitors and staff at the Paris-Descartes University campus.

### Apparatus

Basalt hammerstones were used for this experiment. Each was specifically selected as having the properties required for hardhammer percussion in Oldowan lithic tool production (corresponding to the lower Paleolithic period between 2.6 Myr to 1.7 Myr ago; see Roche, 2005 for further detail). The task analyzed for the purposes of this study involved the use of hammerstones that were roughly ovoid in shape. Flint stone cores served as the raw material for stone flake production. Having been acquired from one unique source, there was limited variability in the quality of the flint itself. All cores had been pre-formed into the shape of a frustrum (a truncated pyramid) by a commercial flint working professional prior to the experiment. This measure served to facilitate immediate flake production by all participants whilst also ensuring that all subjects started under relatively similar conditions.

Movement parameters were recorded using a spatial tracking system (Polhemus Liberty, Polhemus Corporation; Colchester, VT—referred to heron as STS), a device which determines position and orientation of its associated sensors relative to a stationary system by means of an electromagnetic field. This STS permits the recording of movements in six degrees of freedom (x,y,z and rotation along the axes, x,y,z). All data was sampled at a frequency of 240 Hz and recorded online using MotionTracker v1.43 (BIOMETRICS France; Gometz-le-Châtel, Île de France).

### Protocol

The experimental protocol used here respected the ethical guidelines of the American Psychological Association (APA). Following the provision of clear information on the conditions of participation, each subject gave their written consent. Prior to commencing the experimental procedure, personal data (e.g., height, weight, age) was collected and anthropometrical features of the striking arm were recorded in order to permit geometric modeling of the upper limb at a later stage.

STS sensors were applied to the striking arm with adhesive tape at the dorsal surface of the hand, the dorsal surface of the lower arm, the lateral aspect of the upper arm and the dorsal aspect of the coracoid process of the scapula, reflecting the protocol used by Biryukova and colleagues (Biryukova et al., 2000; Biryukova and Bril, 2008) (see Figure 5).

The final stage of preparation involved using the STS stylus to record the location of various anatomical landmarks of the upper limb and thorax in relation to the STS sensors, following the calibrated anatomical system technique (CAST; Cappozzo et al., 1995) and in accordance with the International Society of Biomechanics (ISB) recommendations on joint coordinate systems (Wu et al., 2005). In addition to the stated anatomical landmarks, working surfaces were also defined. This was done by using the STS stylus to record the striking surface of the frustrum in relation to an STS sensor fixed at its base. Similarly, the point of impact used on each hammerstone was recorded in relation to the STS sensor fixed to each subject's striking hand as it was held during the habitual striking grasp.

All subjects received the same instructions and model stone flakes (small and large) were provided to subjects in order to demonstrate the general dimensions of the desired end product. No restriction was placed upon the subjects' seated posture and no time was imposed for completion of the task, allowing subjects to freely explore the materials at hand. Each subject was then required to carry out a series of flaking tasks in a total of six conditions (small and large flake production with hammers of three various masses) in order to determine level of expertise (Bril et al., 2010). Three strikes only were permitted in an attempt to produce one stone flake and subjects were requested to produce three flakes in each condition. All stone flakes removed during this process were collected, weighed and labeled. The image of an expert subject carry out a stone flaking task is provided in Figure 3C.

### Allocation of skill level

**Figure 4:**
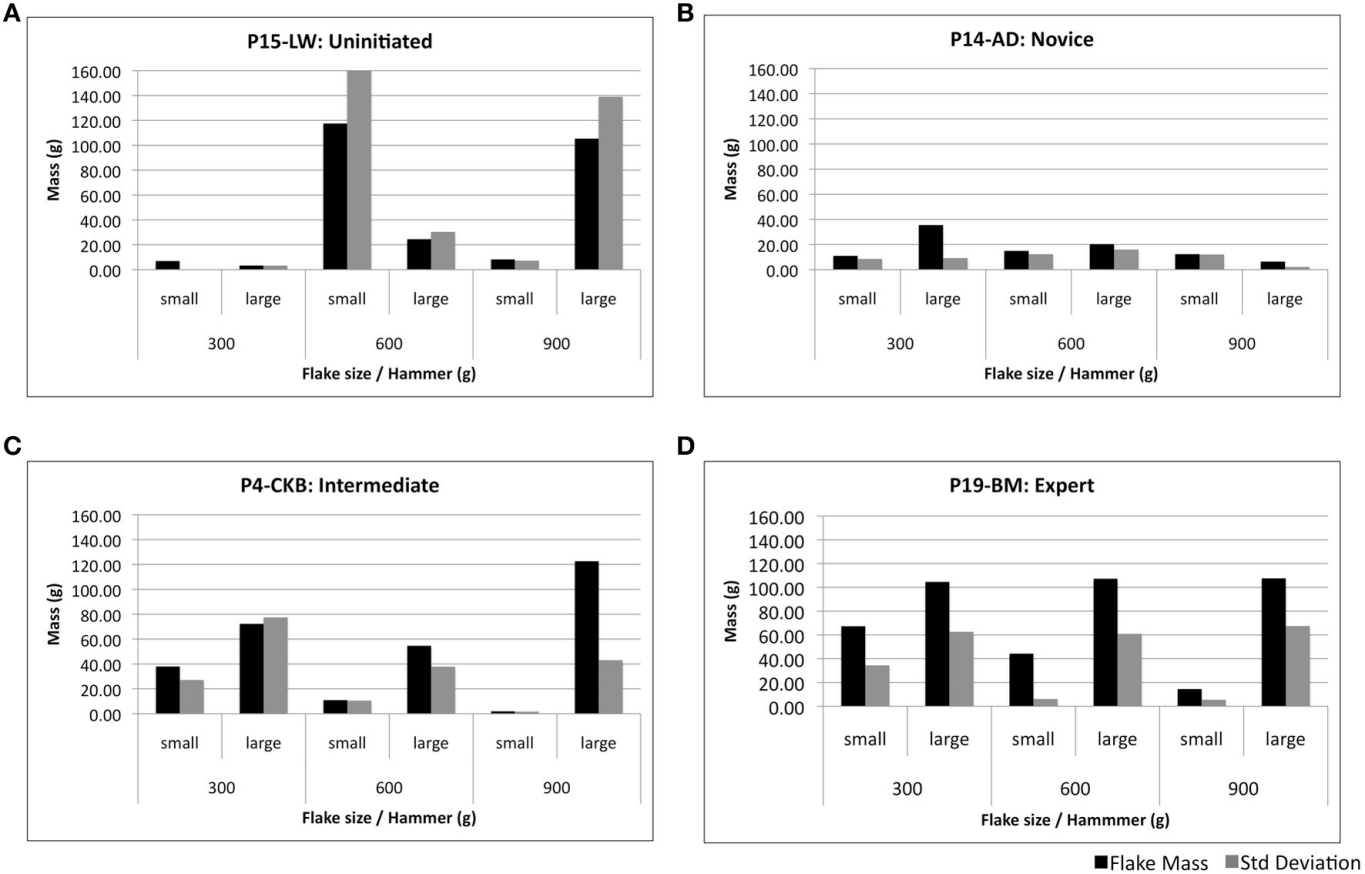
**Samples of task performance profiles used to validate level of expertise**. An uninitiated subject **(A)** demonstrates limited ability to control flake mass. A typical novice subject **(B)** produces more regular flakes but has difficulty to produce flakes of the required size. Intermediate **(C)** and expert **(D)** subjects are respectively more able to produce flakes of the desired dimensions and adapt more easily to hammers of various masses.

Each individual's level of expertise was next attributed based upon their ability to produce small and large flakes with a series of different tools (per Protocol). No qualitative distinction was made to classify the form of a flake as being characteristic of conchoidal fracture or split breaking. Measures of mean flake mass and standard deviation (SD) were used as the basis to determine one's ability to intentionally and consistently control stone flake dimensions, as per Figure 4.

Allocation to both intermediate and expert groups was dependent upon the ability to consistently produce both small and large flakes upon command. Expert status was then attributed to those individuals who produced flakes with a high level of regularity—as indicated by mean flake masses relative to SD.

Novices were defined as subjects with irregular stone flake production. This included firstly those subjects who produced small and large flakes on an inconsistent basis and; secondly, subjects who removed flakes of regular mass and dimension, incapable of producing flakes of varying size during the experiment. Subjects of the uninitiated group were unable to produce flakes of the prescribed size, having highly variable flake masses over the different conditions. The final composition of each group is presented in Table 1.

**Table 1:** **Allocation of subjects to groups according to level of skill**.

**Group**	**Subjects**	**Total**
Uninitiated	P1-SK, P2-NR, P5-SN, P9-CL, P15-LW, P17-RR	6
Novice	P3-SM, P6-SS, P8-TP, P10-ED, P11-SP, P14-AD	6
Intermediate	P4-CKB, P7-AG, P12-OT	3
Expert	P13-LK, P16-CS, P18-JL, P19-BM	4

### Biomechanical modeling

**Figure 5:**
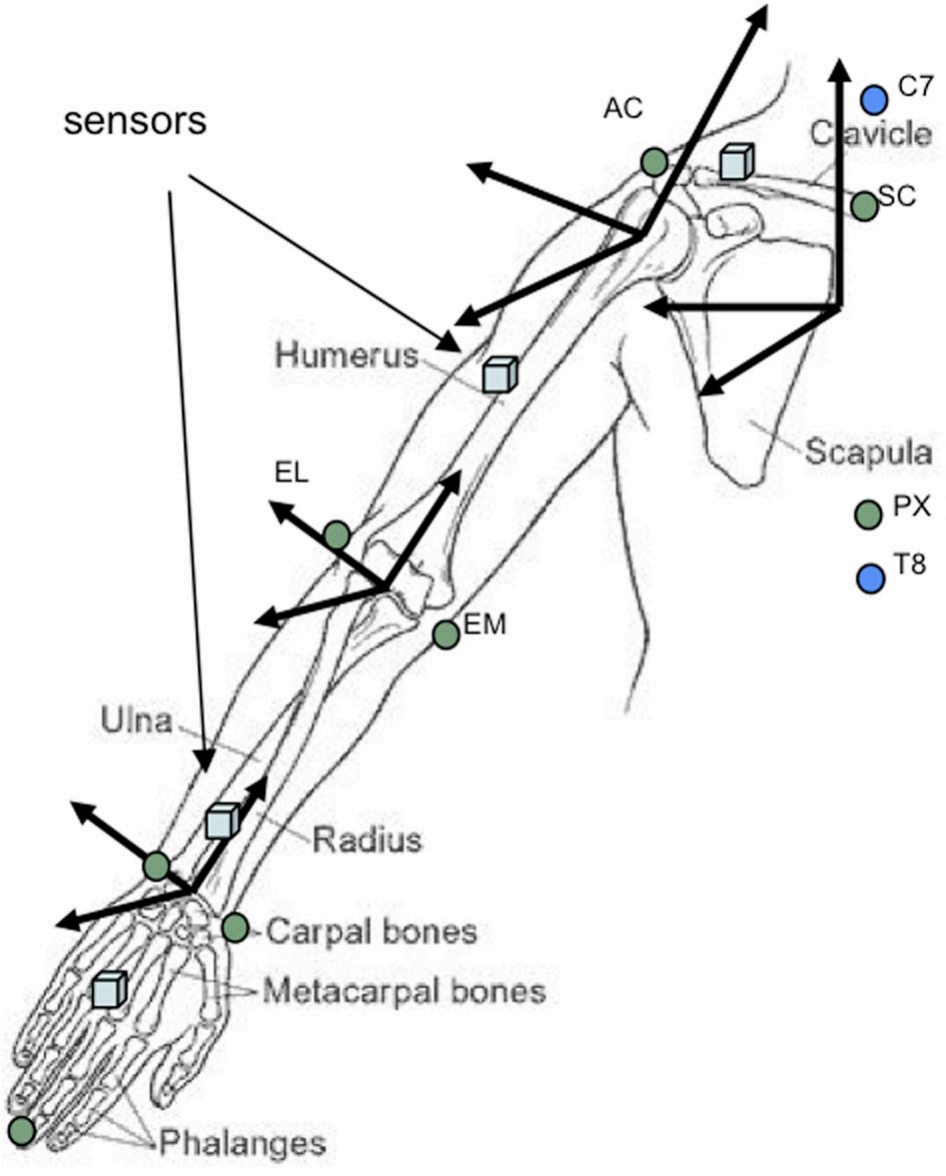
**Location of upper limb movement sensors and calculation of joints using the CAST method (calibrated anatomical system technique)**. Rectangular blue markers correspond to the approximate placement of movement sensors whilst circles indicate anatomical landmarks recorded using the stylus of the spatial tracking system.

The first stages of biomechanical modeling involved the creation of the anatomical frame of reference by calculating the offsets of the anatomical landmarks (recorded with the STS stylus) from the adjacent sensors (refer to Figure 5). The geometric model of the arm, drawn from the manually measured anthropometrical features, was then integrated using the method described by Hanavan (1964). With upper limb and thorax positions in place, the offset of the shoulder joint center from the acromion process was calculated using the sphere fitting process (Leardini et al., 1999; Stokdijk et al., 2000). Following this, elbow and wrist joint centers were calculated based upon the assumption that all joint centers could be found at the center of the axes constructed, respectively, by the two epicondyles at the elbow and the two styloid processes at the wrist (see Figure 5). This method provided one unique joint center at each articulation around which joint axes could then be calculated (Grood and Suntay, 1983; Zatsiorsky, 1998).

An optimization process was then used to eliminate artifacts in the movement data, evident in the form of temporal variations in the distance between adjacent joint centers—a consequence of STS sensor displacement relative to the underlying anatomical landmark and usually the consequence of the deformation of skin and underlying muscular and adipose tissues during the rapid, forceful movement characteristic of percussive tasks. This process involved the recalculation of segment lengths for each subject from sequential frames during a sedentary period of recorded movement data (Lu and O'Connor, 1999; Roux et al., 2002). These recalculated segment lengths were then imposed upon the axes already in place. The final model presented here presents movement relative to three degrees of freedom at each joint for a total of nine degrees of freedom. All movement analysis was performed using customized scripts which were coded using MotionInspector v1.43 (BIOMETRICS France; Gometz-le-Châtel, Île de France).

### Analysis of task performance

Only one specific flaking task was analyzed for the purposes of the present study, that of small flake production with a 600 g hammerstone. This measure ensured that the striking action analyzed corresponded to reasonably equivalent task constraints. This particular task was chosen firstly because most knappers typically prefer hammerstones of this approximate weight and secondly because small flakes tend to be easier to produce than larger flakes (Bril et al., 2010). Measures of flake mass variability were used to determine each subject's ability to intentionally and consistently control stone flake production. These measures included range, SD, and coefficient of variation (CV = SD/mass × 100). The statistical significance of CV between each group was calculated using one way *t*-tests with a Bonferroni correction (*p* = 0.05/6) to determine if CV reduced as a function of expertise.

### Regulation of functional parameters

Relationships between key functional parameters were calculated using data extracted from the biomechanical model. Maximal kinetic energy was determined with reference to the working point of the hammer according to the formula *E*_*k*_ = 1/2 *mv*^2^. Potential energy was calculated with respect to vertical distances between the frustrum and the working point of the tool according to the formula *E*_*p*_ = *mgh* (*g* = 9.81 ms^−2^). Ratios of kinetic energy to potential energy (*E_k_/E_p_*) were also calculated in order to highlight movement strategies in terms of muscular effort (refer to Figure 2).

Two way *t*-tests were used to determine if statistically significant differences existed between the four groups in terms of maximal kinetic energy, potential energy, and the ratio of kinetic energy to potential energy. The Bonferroni correction described above was used in all cases. Whilst data relating to the angle of blow and point of percussion may be extracted from the task specific biomechanical model presented here, this is unfortunately outside the scope of the present paper.

### Postural organization and movement analysis

Video recordings of flaking tasks were synchronized with their corresponding biomechanical reconstruction and permitted an initial qualitative evaluation of joint coordination profiles. The striking movement itself was determined according the displacement of the STS sensor fixed to the striking hand. The beginning of the movement was defined as the moment where the sensor reached its highest point on the vertical axis prior to the strike. The movement was deemed to have ended when the STS sensor reached either its lowest point or when it slowed to a speed inferior to 4.17 × 10^−3^ m/s (the time between two frames at 240 Hz).

### Analysis of coordination by segmental contribution

Principal component analysis (PCA), one of the more classic statistically driven techniques for recognizing patterns in movement data was applied to kinematic data of striking movements. This method was chosen for two reasons. Firstly the PCA facilitated the comparison of movements through compression of the multidimensional datasets. Secondly, use of the covariation matrix served to represent the data in a way that reflects the underlying movement synergies (Ting and Chvatal, 2011). The PCA was applied to each striking movement, following the equation φ_*i*_(*t*) − φ_*Mi*_(*t*) = ∑_*k*_*w*_*ki*_ξ_*k*_(*t*), where the vector of temporal variation of joint angles around their mean values is defined as being equal to the sum of the principal components. Using this mathematical technique, each principal component is presented according to its magnitude so that the first principal component (PC1) corresponds with the axis along which the dataset is most spread; the second principal component (PC2) describes an orthogonal axis which describes the next most important data variance and so on. Use of this method generally permits the description and analysis of a significant percentage of movement data through one or two matrices (Rein, 2012).

Finally, the regularity of joint angle contributions to PC1 was then calculated for each subject's three successful attempts. This was done using by comparing absolute values of Pearson's correlation coefficient from the PC1 loadings across each of the nine degrees of freedom represented in the biomechanical model strikes (successful strike 1 vs. successful strike 2; successful strike 1 vs. successful strike 3; successful strike 2 vs. successful strike 3). The correlation coefficient was considered to be significant at a value greater than 0.7.

## Results

### Task performance

The data pertaining to the specific task of small flake production with the 600 g hammerstone yielded a total of 89 strikes, of which 53 produced a flake. From the three attempts granted to each subject per trial, uninitiated subjects employed an average of 2.2 strikes in order to remove a flake while novices used an average of 1.6 strikes. Intermediate and expert group subjects used an average 1.3 and 1.4 strikes per trial respectively in removal of the stone flakes.

Given that successfully controlling the size of stone flake production was the goal of the set task, flake mass variability was used as the primary indicator of task performance. Summary statistics of flake mass production by group are provided in Table 2. Overall, uninitiated subjects produced flakes with highly variable results (*SD* = 65.1 g; range = 1–232 g), as did members of the novice group (*SD* = 30.5 g; range = 2–94 g). The intermediate (*SD* = 23.7 g; range = 1–75 g) and expert groups (*SD* = 19.7 g; range = 2–56 g) were more consistent in their stone flake production. Importantly, SD and range of the mass of flakes produced by each group can be seen to decrease according to the level of expertise (as can be seen in Table 2). It is also interesting to note that the median flake mass of the expert group is the same as the model flake provided (12 g).

**Table 2:** **Summary statistics of flake mass and regulation of kinetic energy by group**.

**Group**	**Avg number of strikes per flake**	**Flake mass (g)**					**Max kinetic energy (j)**		**Max potential energy (j)**	
		**Mean**	**Median**	***SD***	**Range**	**Avg CV**	**Mean**	***SD***	**Mean**	***SD***
Uninitiated	2.2	28.6	3.5	65.1	1–232	103	12.30	4.88	3.39	1.06
Novice	1.6	29.9	10	30.5	2–94	89	4.20	1.27	2.24	0.47
Intermediate	1.3	19.3	8	23.7	1–75	98	5.21	2.36	2.13	1.34
Expert	1.4	22	12	19.7	2–56	64	4.44	2.56	1.37	0.45

No statistically significant difference of CVs between the respective groups was found following *t*-tests. It should be recognized however that the power of any statistical test would be limited given the small sample sizes.

### Regulation of functional parameters

Novice, intermediate and expert subjects all produced relatively similar levels of kinetic energy across the task in question (mean *E_k_* = 4.20 J, *SD* = 1.27 J; mean *E_k_* = 5.21 J, *SD* = 2.36 J; mean *E_k_* = 4.44 J, *SD* = 2.56 J, respectively). Uninitiated subjects demonstrated exceptionally high levels of kinetic energy, at an average of 12.30 J (*SD* = 4.88 J), a factor which proved to be statistically significant to all other groups.

Similarly, potential energy upon initiation of the striking movement was also particularly high amongst subjects of the uninitiated group (*E_p_* = 3.39 J, *SD* = 1.06 J) and was again statistically significant using two tailed *t*-tests. Mean values for potential energy did however show a tendency to decrease as a function of expertise with mean values of 2.24 J (*SD* = 0.47 J) for the novice group, 2.13 J (*SD* = 1.34 J) for the intermediate group and 1.37 J (*SD* = 0.45 J) for the expert group. This difference proved to be statistically significant between the novice and expert groups.

**Figure 6:**
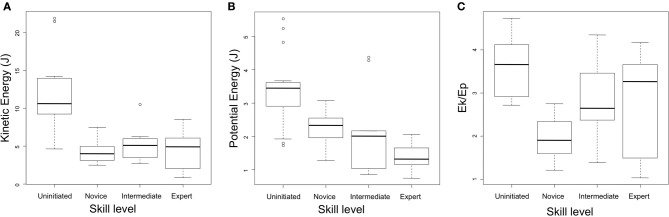
**Regulation of functional parameters according to level of expertise. (A)** Mean values of maximal kinetic energy of the hammer. **(B)** Potential energy relative to the striking surface of the core. **(C)** Ratios of kinetic energy to potential energy. Whisker values indicate the confidence interval at 0.95.

The average ratio of kinetic energy to potential energy was also very high amongst uninitiated subjects (mean *E_k_/E_p_* = 3.64, *SD* = 0.82). Conversely, novice subjects demonstrated particularly low average ratios of kinetic energy to potential energy (*E_k_/E_p_* = 1.90, *SD* = 0.50), with intermediate and expert subjects demonstrating respectively higher ratios of mean kinetic energy to potential energy (*E_k_/E_p_* = 2.82, *SD* = 0.94 and *E_k_/E_p_* = 3.04, *SD* = 1.39, respectively). Multiple *t*-tests with Bonferroni corrections proved all groups to be significantly different to each other. Data on the regulation of functional parameters is presented in both Table 2 and Figure 6.

### Postural organization and movement analysis

Individual differences in terms of postural preference and movement profiles were evident upon analysis of video data synchronized with the reconstructed kinematic model. Some subjects positioned the core upon or against their leg whilst other subjects held the core in front of their body (see also Bril et al., 2010). Whilst the majority of subjects carried out the task whilst seated on a stool, certain subjects chose to be seated on the ground for the duration of the flaking tasks.

**Figure 7:**
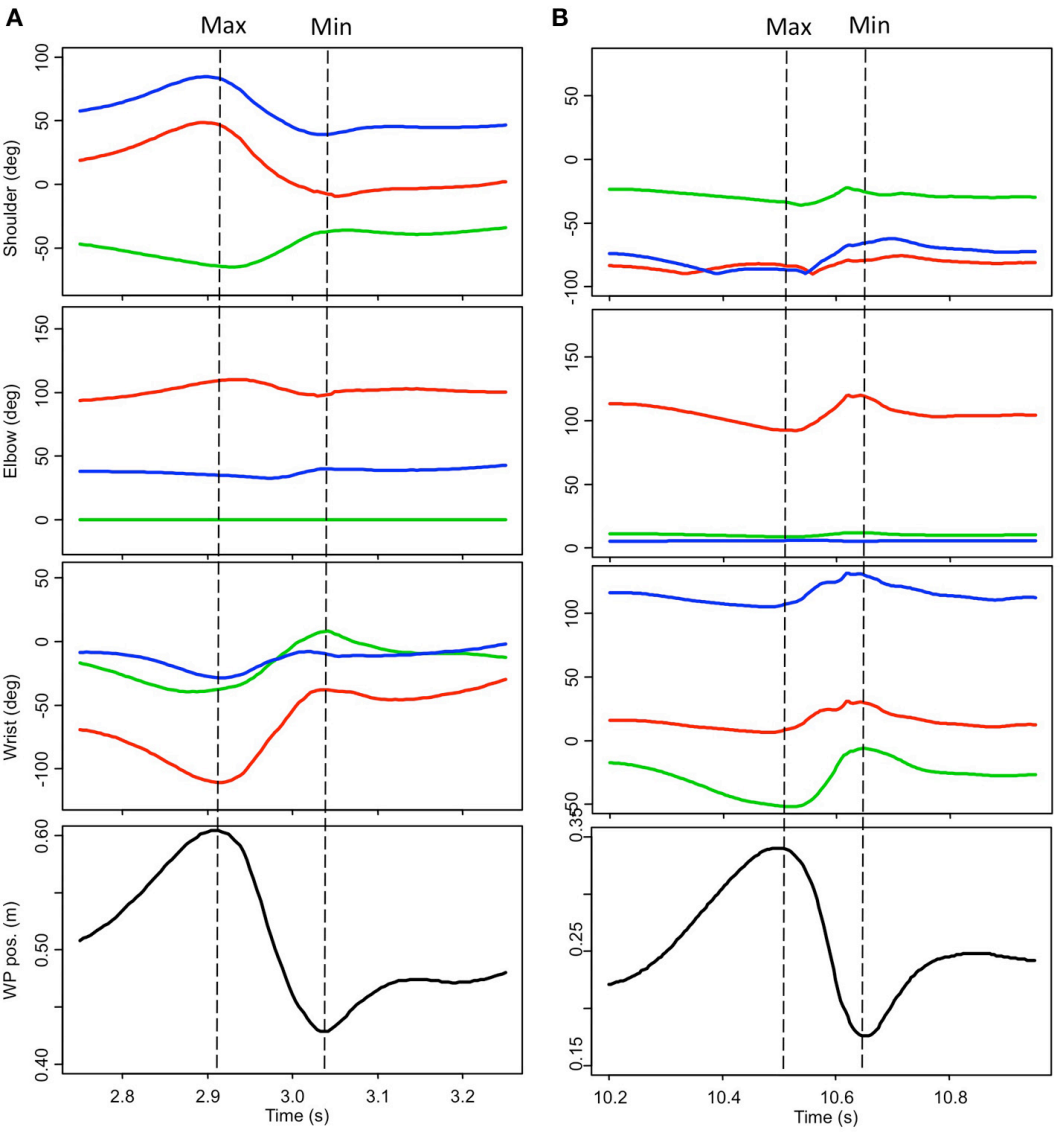
**Angular variations of the striking arm for two expert stone knappers during the successful removal of a stone flake. (A)** P18-JL strike 2. **(B)** P19-BM strike 2. All joint values are given in degrees; Flex/Ext (red); Add/Abd (green); Rot Int/Ext (blue). Movement of the working point along the vertical axis is depicted in the bottom-most panel. No data presented here has been filtered. Max and min indicated the beginning and the end of the striking movements themselves.

Spatiotemporal aspects of striking movements were also seen to be vary both on intraindividual and interindividual bases. Some subjects demonstrated striking movements characterized by high levels of wrist contribution, other subjects demonstrated movements characterized by high levels of elbow contribution. No apparent relationship between kinematic movement organization and expertise was evident. An example of two expert strikes (P18-JL strike 2 and P19-BM strike 2) is provided in Figure 7. It is interesting to note that while angular variations and the duration of the striking movements are quite different in these two strikes, the working point trajectories appear remarkably similar.

### Joint angle contribution

Overall, PC1 accounted for 71% of joint angle variation (median = 72%; *SD* = 8%; range 55–81%), while PC2 accounted for 17% (median = 18%; *SD* = 5%; range = 11–25%) of joint variation in the flaking task. The percentage of joint angle variation accounted for by PC1 in the uninitiated group members was observed to be considerably lower than that of other groups at 64%. Limited differences were evident in the percentages of joint angle variation accounted for by PC1 in the other three groups, with PC1 accounting for 74, 75, and 76%, respectively, for the novice, intermediate and expert groups. PC1 and PC2 combined accounted for greater than 90% of joint angle variation in all groups except for the uninitiated group for whom the sum of PC1 and PC2 accounted for 84%. Having this amount of variance expressed by the first two principal components is indicative of a level of compression sufficient for valid data analysis (Rein, 2012).

**Figure 8:**
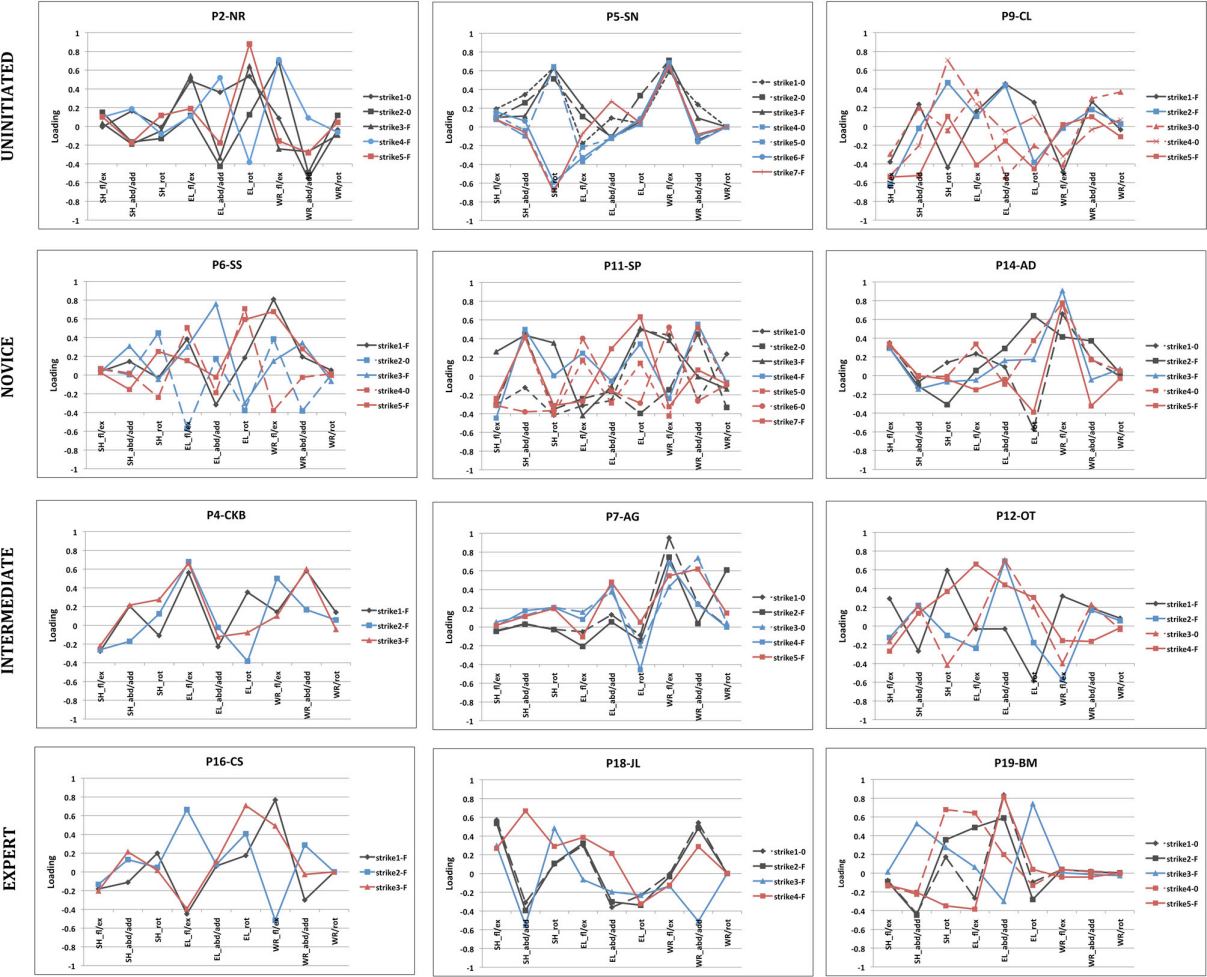
**Joint angle contributions to the first principle component**. A sample of strikes used in the production of three small flakes with a 600 g hammerstone. Three subjects from each skill level group (uninitiated, novice, intermediate, and expert) are provided as examples. Black traces represent attempts made to produce the first flake, blue traces represent attempts used when making the second flake and red traces for the third flake. Strikes that produce flakes are indicated with an “F,” strikes that failed to produce a flake are indicated with a “0.”

The loading factors of PC1 and PC2 were used to analyze relative joint contributions to each movement and the regularity of coordinative structure in each subject's attempts at the task in question. Figure 8 provides joint angle loadings on PC1 for three subjects representative of each group. This Figure highlights the exceptional variability of movement strategies employed by the subjects of this experiment, on both interindividual and intraindividual bases.

The variability of coordinative structure in strikes that successfully removed a flake was also examined on an individual basis using Pearson's correlation coefficients (these strikes are indicated by an “F” in Figure 8). No subject had joint angle loadings with significant correlation coefficients in all three comparisons. These results indicate that successful flake production was not dependent upon consistent patterns of joint angle coordination.

## Discussion

This study examined tool use actions in a healthy adult population by means of kinematic movement analysis. From the outset, it was proposed that tool use capacity was based upon a learned ability to manipulate the functional dynamics of a given situation, as opposed to being a skill determined by movement characteristics *per se*. As such, we expected individuals to demonstrate a variety of motor patterns during a functionally equivalent (having the same task constraints) tool use activity. Using stone flake production by hardhammer percussion (based upon the Oldowan tradition) as the tool use activity in the experimental procedure, this study included participants of varying levels of experience at the task; from those with no prior exposure to the activity, through to individuals with many years of regular practice.

Relationships between the regulation of functional parameters and movement parameters (see Figure 2) were observed in these four different groups. We hypothesized that given the task constraints, countless combinations of kinematic patterns may constitute a viable action, capable of producing the desired result. That is to say, we proposed that no particular kinematic movement pattern would be necessary or characteristic of successful task performance. In addition to this, it was hypothesized that the variability of a subject's joint angle contributions in the striking action would vary as a function of expertise. Specifically, we expected that uninitiated group subjects would use varied combinations of movements as they explored functional dynamics while novice and intermediate subjects would have comparably less movement variability, indicative of the limited number of task-specific movement patterns in their motor repertoire. Finally, movement variability was expected to be relatively high in experts, as robust synergies would exploit the multiple degrees of freedom at play in the body-tool-environment system in order to stabilize the functional parameters of the task.

**Figure 9:**
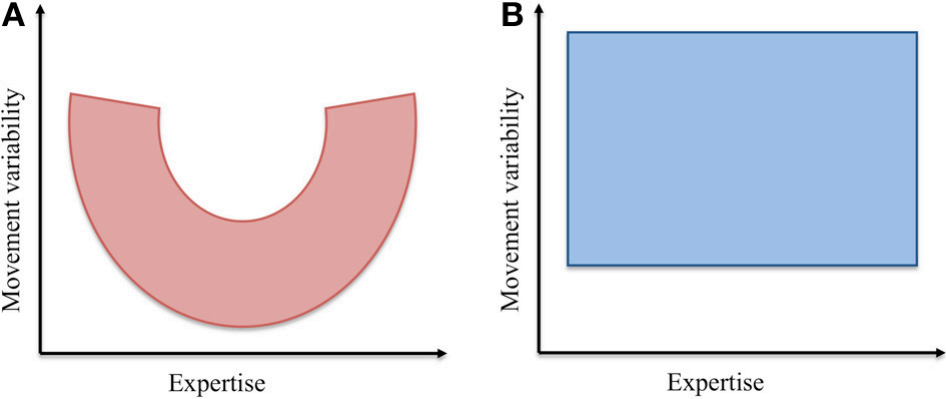
**Schematic representations of the hypothesized and the observed relationships between movement variability and expertise**.

The results of this experiment proved the first of these hypotheses to be correct; successful task performance was not correlated with any specific movement pattern. The second of these hypotheses proved incorrect. Indeed, both subjects with highly variable joint contribution profiles and subjects with comparably less variable joint contribution profiles were present in each skill level group (see Figures 8, 9). These particular results hold interest firstly in the context of the existing body of work on stone knapping and skill acquisition in early man; and secondly, in the broader context of understanding the cognitive and neurological bases of human tool use.

The analysis of the kinematic movement data presented here revealed that successfully removing stone flakes from a flint core by the Oldowan method was not contingent upon specific contributions of the wrist, elbow or shoulder. Effective stone flaking actions characterized by relatively high contributions of each joint were observed through the course of the experiment (see Figure 8, P7-AG; P4-CKB; and P18-JL for respective examples). This result may be seen to validate previously contrasting findings of experiments that found stone flaking actions to be characterized by high levels of wrist contribution (Williams et al., 2010) and others finding the elbow to have the most important contribution to the action (Rein et al., 2013).

Although based upon a different technique, the highly individual nature of upper limb kinematics demonstrated by the subjects in this study reflects the findings of Biryukova and colleagues (Biryukova and Bril, 2008; Biryukova et al., in press). Further to this, the results of the present study also indicate that patterns of kinematic movement variability are not a reliable measure of skill (cf. Biryukova et al., in press). Rather, relative tool use ability—expertise as it were, was manifested principally by the stability and intentional control of task performance. Further differences between those with no task specific experience (uninitiated) through to expert practitioners of the stone knapping technique were also apparent through the regulation of and sensitivity to key functional parameters (Bril et al., 2010). Relationships between these different parameters is highlighted here in Figure 2 and facilitates the analysis of tool use ability in terms of action.

With respect to actual stone flake production, members of the uninitiated group demonstrated particularly erratic performance as evidenced by high measures of statistical dispersion. This variability of stone flake production (indicated by flake mass) was generally observed to reduce with each respective level of expertise, as shown by the corresponding reduction in range and SD of flake mass. Expecting identical flake dimensions upon successive strikes is of course unrealistic as even despite pre-shaping, each core varied slightly in terms of form, reflective of a real life situation.

The values of kinetic energy produced by members of the respective groups was coherent with the characteristics of the flakes produced. Subjects of the uninitiated group exhibited exceptionally high levels of kinetic energy, signifying their lack of understanding in fracture mechanics. Whilst no qualitative classification of stone flakes was conducted to discern between split breaking and conchoidal fracture in this experiment, it may safely be assumed that such amounts of kinetic energy would typically have been in excess of the threshold at which split breaking occurs, thereby producing flakes of highly variable dimensions. In contrast, the values of kinetic energy produced by novice group members is approximately half that of the uninitiated group. One may infer that these subjects, having already been inducted into basic stone knapping techniques, possessed a sound awareness of managing this key functional parameter when attempting to control stone flake dimensions. Although having somewhat greater ranges, the intermediate and expert groups produced mean values of kinetic energy similar to that of the novice group. It is interesting to note however, that expert subjects tended to produce larger flakes on average than their intermediate counterparts (as indicated by mean and median flake mass, see Table 2). The utilization of more energy efficient motor solutions exhibited by expert subjects here reflects those findings of Nonaka et al. (2010). It suggests that beyond an appreciation of kinetic energy, higher-level stone knappers have a greater appreciation of the nested relationships between existing between the angle of blow, point of percussion, and external angle (see Figures 2, 3).

A general trend in the use of potential energy is also evident between the four skill level groups. As can be observed in Figure 6B, subjects tend to reduce the amplitude of their movements (and in such a way the amount to which they harness gravity in generating the necessary energy for stone flake removal) as expertise increases. In the case of the uninitiated group, generating large amounts of potential energy is clearly representative of their limited understanding of task constraints (per above). The ratios of kinetic energy to potential energy shown in Figure 6C give further insights into these movement strategies and indicate an increase in the use of muscular energy from novices to experts. The reason for this trend however, is not clearly evident. It may be position that this effect is simply a reflection of confidence, whereby subjects who are sure of their actions employ less ample movement but with notably greater velocity. Or it could be that this strategy is of functional importance, and that the greater levels of acceleration (deduced here from the relationship between resultant velocity and length of hammer trajectory) may improve propagation of the shock involved in conchoidal fracture, reducing the likelihood of step fractures or the production of other undesirable features upon the core.

As opposed to task performance and the regulation of functional parameters, kinematic aspects of upper limb movement in terms of joint angle contribution were not capable of distinguishing subjects of different levels of tool use ability. Subjects with highly variable movement patterns and subjects with comparably regular movement patterns were present in each of the four skill level groups. Furthermore, it is also interesting to note that on several occasions, two movements with quite similar PC1 loadings produced different results. For example, P18-JL is shown to employ movements with almost identical PC1 loadings on both his first and second strikes (see Figure 8), but whilst the first is unsuccessful, the second strike successfully produces a flake. Of course, what cannot be discerned from the present analysis, is if and how this subject may have adjusted other factors, such as the orientation of the core at the time of the strike. Again, one cannot truly determine the efficacy of the movement independently of the tool-environment system with which it must be synchronized.

This finding that the regularity of kinematic movement patterns does not correspond with levels of expertise in this percussive tool use activity may initially appear to be contrary to intuition. Indeed, movement variability is often (incorrectly) perceived as being related to error, and is typically thought to decrease with improved task performance (Stergiou and Decker, 2011). From a strictly mechanical perspective however, countless combinations of joint angle contributions may produce viable motor solutions to a given problem. The considerable variability of movement patterns demonstrated in and across each skill level group in this experiment support our assertion that during tool use, the nervous system learns to manage the action rather than the movement. In any case, perfect reproduction of a certain movement in no way affords the possibility of adaptable behavior. Instead, it seems apparent that humans learn to improve the effects of their actions by increasing their understanding of functional dynamics. And rather than a hindrance, this variability witnessed in patterns of joint angle coordination could be seen to enhance this process of motor learning (Wu et al., 2014).

At the outset of this experiment, the hypothesis regarding the evolution of movement variability and expertise was founded upon this idea of exploring, then exploiting the mechanical properties comprising the body-tool-environment system. In addition, we contended that synergies provided a viable means for the neural control of functional dynamics during dexterous tool use activities. The experimental results presented here did not however, reflect a varying composition of coordinative structures across the skill level continuum (see Figures 8, 9). Despite this outcome, we do not interpret this result as meaning that individuals of all skill levels possess equally flexible families of task specific motor solutions. The simple fact is that predicting whether movement variability is a good or a bad thing is not a straightforward matter. In the study of Rein et al. (2013) for example, novice subjects actually exhibited greater magnitudes of compensated variability of working point position than their expert counterparts—the effect of this was just negated by a level of uncompensated variability that was also higher than that of the experts.

In the present study, certain differences in joint contribution profiles appear simply to reflect changes in the positioning of the core, adjustments of tool grip or other such postural preference. The causes of these postural variations may in effect have limited relation to an individual's level of expertise; to maintain exactly the same posture over any duration of time is, under normal circumstances, not only uncommon but rather challenging in itself. In order to observe the highly adaptable movement synergies that would distinguish novice subjects from expert subjects, it is likely that more challenging conditions would need to be introduced to the experimental protocol (e.g., using a different tool, performing under stress/fatigue, responding to external perturbations).

In conclusion, this study has found that tool use ability depends primarily upon an understanding of the functional dynamics that exist across the body-tool-environment system, and that kinematic movement profiles alone are not sufficient to indicate relative skill levels. In other words, when learning a tool use activity, what the individual learns is the functional dynamics of the task rather than any particular movement *per se*. We argue that functional approaches such as the one employed here are imperative to the understanding of goal directed activity in cognitive neuroscience. Logically, it must first be understood what a person is controlling in terms of task relevant parameters in order to understand that which is being represented by the brain.

### Conflict of interest statement

The authors declare that the research was conducted in the absence of any commercial or financial relationships that could be construed as a potential conflict of interest.
